# Identifying Risk Factors for Recent HIV Infection in Kenya Using a Recent Infection Testing Algorithm: Results from a Nationally Representative Population-Based Survey

**DOI:** 10.1371/journal.pone.0155498

**Published:** 2016-05-19

**Authors:** Andrea A. Kim, Bharat S. Parekh, Mamo Umuro, Tura Galgalo, Rebecca Bunnell, Ernest Makokha, Trudy Dobbs, Patrick Murithi, Nicholas Muraguri, Kevin M. De Cock, Jonathan Mermin, 2007 KAIS study group

**Affiliations:** 1 US Centers for Disease Control and Prevention (CDC), Center for Global Health (CGH), Division of Global HIV and Tuberculosis (DGHT), Nairobi, Kenya; 2 US CDC, CGH, DGHT, Atlanta, Georgia, United States of America; 3 Kenya Ministry of Health, National Public Health Laboratory Services, Nairobi, Kenya; 4 Kenya Ministry of Health, National AIDS Control Council, Nairobi, Kenya; 5 Kenya Ministry of Health, National AIDS and STI Control Programme, Nairobi, Kenya; 6 US CDC, CGH, Nairobi, Kenya; University of Pittsburgh, UNITED STATES

## Abstract

**Introduction:**

A recent infection testing algorithm (RITA) that can distinguish recent from long-standing HIV infection can be applied to nationally representative population-based surveys to characterize and identify risk factors for recent infection in a country.

**Materials and Methods:**

We applied a RITA using the Limiting Antigen Avidity Enzyme Immunoassay (LAg) on stored HIV-positive samples from the 2007 Kenya AIDS Indicator Survey. The case definition for recent infection included testing recent on LAg and having no evidence of antiretroviral therapy use. Multivariate analysis was conducted to determine factors associated with recent and long-standing infection compared to HIV-uninfected persons. All estimates were weighted to adjust for sampling probability and nonresponse.

**Results:**

Of 1,025 HIV-antibody-positive specimens, 64 (6.2%) met the case definition for recent infection and 961 (93.8%) met the case definition for long-standing infection. Compared to HIV-uninfected individuals, factors associated with higher adjusted odds of recent infection were living in Nairobi (adjusted odds ratio [AOR] 11.37; confidence interval [CI] 2.64–48.87) and Nyanza (AOR 4.55; CI 1.39–14.89) provinces compared to Western province; being widowed (AOR 8.04; CI 1.42–45.50) or currently married (AOR 6.42; CI 1.55–26.58) compared to being never married; having had ≥ 2 sexual partners in the last year (AOR 2.86; CI 1.51–5.41); not using a condom at last sex in the past year (AOR 1.61; CI 1.34–1.93); reporting a sexually transmitted infection (STI) diagnosis or symptoms of STI in the past year (AOR 1.97; CI 1.05–8.37); and being aged <30 years with: 1) HSV-2 infection (AOR 8.84; CI 2.62–29.85), 2) male genital ulcer disease (AOR 8.70; CI 2.36–32.08), or 3) lack of male circumcision (AOR 17.83; CI 2.19–144.90). Compared to HIV-uninfected persons, factors associated with higher adjusted odds of long-standing infection included living in Coast (AOR 1.55; CI 1.04–2.32) and Nyanza (AOR 2.33; CI 1.67–3.25) provinces compared to Western province; being separated/divorced (AOR 1.87; CI 1.16–3.01) or widowed (AOR 2.83; CI 1.78–4.45) compared to being never married; having ever used a condom (AOR 1.61; CI 1.34–1.93); and having a STI diagnosis or symptoms of STI in the past year (AOR 1.89; CI 1.20–2.97). Factors associated with lower adjusted odds of long-standing infection included using a condom at last sex in the past year (AOR 0.47; CI 0.36–0.61), having no HSV2-infection at aged <30 years (AOR 0.38; CI 0.20–0.75) or being an uncircumcised male aged <30 years (AOR 0.30; CI 0.15–0.61).

**Conclusion:**

We identified factors associated with increased risk of recent and longstanding HIV infection using a RITA applied to blood specimens collected in a nationally representative survey. Though some false-recent cases may have been present in our sample, the correlates of recent infection identified were epidemiologically and biologically plausible. These methods can be used as a model for other countries with similar epidemics to inform targeted combination prevention strategies aimed to drastically decrease new infections in the population.

## Introduction

Over the past decade, substantial scale-up of HIV care and treatment programs globally has resulted in approximately 16 million people accessing HIV treatment by 2015 [1]. Yet antiretroviral treatment (ART) coverage has not reached universal access in most resource-constrained countries, and other HIV prevention efforts have not sufficiently reduced HIV transmission and new infections. Sub-Saharan Africa remains disproportionately affected by the HIV epidemic, accounting for 70% of HIV infections worldwide, 70% of new HIV infections among adults, and 86% of new HIV infections among children [1]. With the implementation of large-scale combination prevention strategies, including voluntary male medical circumcision and intensified access to treatment [2–7], accurate tools to monitor where and among whom new HIV infections are occurring are essential to evaluate the impact of these strategies and to improve the efficiency of targeted prevention programs.

There are several approaches for measuring the occurrence of new HIV infections, the gold standard being measurement through prospective cohorts that track HIV seroconversion among HIV-uninfected persons. The most common approach in developing nations, however, has been through inference, using trends in HIV prevalence and assumptions about HIV-specific mortality and the impact of antiretroviral therapy (ART) coverage on survival [8–11]. While these indirect measures have been useful in estimating HIV incidence across important demographic strata, they cannot provide information beyond these basic metrics, limiting their utility for targeted programming.

Several groups have developed laboratory-based approaches to distinguish recently acquired HIV infections from HIV infections of longer duration in cross-sectional surveys [12–14]. HIV incidence assays are based on the underlying principle that the immunological response to HIV infection evolves for a number of months following infection, allowing for the identification of immunological biomarkers of early HIV disease progression that can serve as indicators of recent infection. If accurate, incidence assays can provide a rapid and cost-effective approach for providing reliable and up-to-date information on the dynamics of HIV transmission for more effective planning [15]. The Limiting Antigen Avidity Enzyme Immunoassay (hereafter termed “LAg”) has been previously described as a tool for estimating population-level HIV incidence in settings world-wide [16–18]. Recent infection testing algorithms (RITA) that combine the results of an HIV incidence assay with markers of chronic infection to confirm recent infection (e.g., antiretroviral therapy use and viral suppression), is the recommended approach for distinguishing recent from long-standing infection in a survey setting [19].

As we move closer towards substantially reversing the HIV epidemic, countries are being called to evaluate the public health impact of intensified scale-up of combination prevention strategies on the prevention of new HIV infections in the population. To demonstrate the utility of RITAs to inform such strategies, we applied a LAg-based RITA to blood specimens collected in a nationally representative serologic survey in Kenya to identify factors associated with recent infection in 2007. These methods were designed to be replicable in countries with similar epidemics and epidemiological data sources to provide insight into sub-populations most vulnerable to incident infection for targeted prevention.

## Materials and Methods

### Study design

The 2007 Kenya AIDS Indicator Survey was a two-stage cluster household survey that produced nationally representative estimates of HIV indicators in Kenya [20]. Participants aged 15–64 years consented to a questionnaire on demographics; behavior; knowledge and attitudes towards HIV/AIDS; and access to care, treatment, and prevention programs for HIV infection. Blood specimens were collected to test for HIV-1 antibodies; CD4 cell counts using Becton, Dickinson and Company (BD) FACSCalibur flow cytometer (BD Biosciences, San Jose, CA), if found to be HIV infected; HSV-2 testing using Kalon HSV Type 2-specific IgG enzyme immunoassay (Kalon Biologicals, Guildford, United Kingdom); and syphilis testing using the Treponema pallidum particle agglutination assay (Serodia-TPPA, Fuirebio Diagnosis Inc, Tokyo, Japan) as the screening assay and confirmed using the rapid plasma regain test (BD, Franklin Lakes, New Jersey, USA) on undiluted serum specimens to test for reaginic antigens. All testing was conducted in a central laboratory in Nairobi, and results were returned to participants approximately 6 weeks after blood collection. Consent was requested from participants for permission to store remnant blood specimens in -70°C freezers at the central laboratory for future unspecified testing. Participants were informed that test results from future tests would not be returned.

### Laboratory methods

Using methods described elsewhere [21], the LAg was applied to stored HIV-antibody-positive serum specimens from survey respondents using a commercialized kit (Sedia Biosciences, Portland, Oregon). Specimens with final normalized optical density (ODn) values >1.5 were classified as non-recent on the assay while those with final ODn values ≤1.5 were classified as recent on the assay [19]. Prior to LAg testing, specimens had been stored in the laboratory freezer for approximately three years.

Approximately seven years after data collection, HIV-antibody positive dried blood spot (DBS) specimens were retrieved from freezer storage and tested for HIV-1 RNA concentration (Abbott M2000 Real-Time HIV-1 Assay, Abbott Laboratories, Abbott Park, IL). Viral load testing resulted in undetectable levels of HIV-1 RNA in over 90% of samples tested. While the lack of viral RNA was suggestive of nucleic acid degradation over the seven year storage period we were unable to confirm this with additional laboratory analysis. As such, viral load testing data were not included in this analysis. Following viral load testing, remaining DBS specimens were transported to the University of Cape Town in South Africa where a qualitative ARV drug assay was applied to specimens to test for the presence of four antiretroviral (ARV) drugs, including nevirapine (NVP), efavirenz (EFV), lamivudine (3TC), and lopinavir (LPV). At the time of the survey, the first-line standardized national ARV drug regimen was stavudine (d4T) or zidovudine (AZT) + 3TC + EFV or NVP, and the second line regimen was didanosine (ddI) + abacavir (ABC) + lopinavir/ritonavir (LPV/r) or tenofovir (TDF) + ABC + LPV/r [22].

ARV drug testing was conducted using a qualitative ARV assay using liquid chromatography-tandem mass spectrometry (MS), a method applied previously in national household surveys in South Africa [23]. The method consisted of incubating the DBS punches (2 x 3.0 mm) in water for 10 minutes, followed by protein precipitation using Acetonitrile and by high performance liquid chromatography with MS/MS detection. Efavirenz-d5, nevirapine-d3, lopinavir-d8 and lamivudine-^15^N_2_,^13^C were used as the internal standards. Chromatographic separation was achieved using a Phenomenex Luna 5μ PFP (2) 50 x 2.00 mm analytical column. An AB Sciex API 4000 mass spectrometer at unit resolution using the Scheduled MRM™ algorithm was used to monitor the transition of the protonated precursor ions m/z 316.0 and m/z 321.0 to the product ions m/z 244.0 and m/z 246.0 for efavirenz and efavirenz-d5, m/z 266.9 and m/z 270. 0 to the product ions m/z 198.2 and m/z 229.1 for nevirapine and nevirapine-d3, m/z 629.4 and m/z 637.4 to the product ions m/z 120.2 and m/z 191.2 for lopinavir and lopinavir-d8, m/z 230.2 and m/z 233.2 to the product ions m/z 112.0 and m/z 115.2 for lamivudine and lamivudine-^15^N_2_,^13^C, respectively. A cut-off standard with a concentration of 0.020 μg/ml was verified against regressions constructed using calibration standards for each analyte. A specimen with levels observed greater or equal to 0.020 μg/ml was designated as positive for the analyte while levels below 0.020 μg/ml were deemed negative for the analyte. Specimens that tested positive for one or more analytes were classified as having the ARV biomarker.

### Classification of recent and long-standing infection

We applied a RITA where the recent infection case definition was defined as: 1) testing recent on LAg and 2) not from an HIV-infected person who was using ART. ART use was defined as either presence of the ARV biomarker or self-reported ART use during the survey interview for respondents whose specimens that were not tested for the ARV biomarker. Long-standing infection was defined as: 1) testing non-recent on LAg or 2) testing recent on LAg and from an HIV-infected person who was taking ART. Because the reported mean duration for recent infection (MDRI) for LAg for predominate HIV-1 subtypes in Kenya varies from 109 days for subtypes A and D infections combined, 172–211 days for subtype A infections, and 273–283 days for subtype D infections [24–26], for the purposes of this analysis recent infection was presumed to represent HIV infection acquired roughly less than one year prior to specimen collection while long-standing infection was presumed to represent HIV infection acquired roughly one year or greater prior to specimen collection.

### Data analysis

Descriptive analyses were conducted to determine the proportion of recent and long-standing infection by select demographic, behavioral, and biological characteristics. The Kruskal-Wallis test was used to test for differences in medians. Variables associated with recent and long-standing infection at a significance level of p-value <0.2 in bivariate analysis and those known to be risk factors for HIV infection or suspected confounders were included in multinomial logistic regression models which used an ordinal scale of the outcome variable where HIV uninfected was classified as outcome category = 1, serving as the referent group; long-standing infection classified as outcome category = 2; and recent infection classified as outcome category = 3 [27]. This categorization allowed the model to identify independent and significant risk factors for recent and long-standing infection simultaneously allowing for direct comparison of the magnitude of the measure of associations reported for recent infection and long-standing infection. A separate multivariate logistic regression model was applied to directly compare recent and longstanding infection with respect to demographic, behavioral, and biological factors. Missing categories were created to account for missing data for female participants for male-specific variables (e.g., male circumcision and presence of male genital sores or ulcers) and male participants for female-specific variables (e.g., current pregnancy based on self-report). Adjusted odds ratios (AOR) and 95% confidence intervals (CI) were presented for the final estimates in the models. Associations with 95% CIs that did not include 1.0 were considered significantly associated with recent or long-standing infection. Data analysis was conducted in SAS version 9.3 (SAS Institute, Cary, NC, USA). All analyses were weighted to account for the complex survey design and adjusted for survey nonresponse.

This study was reviewed and approved by the institutional review boards (IRBs) at the Kenya Medical Research Institute (KEMRI) and the US Centers for Disease Control and Prevention (CDC). As standard practice for surveys in Kenya, verbal informed consent with a signature of the interviewer as documentation of consent was obtained for adult participants aged 18–64 years. For adolescents aged 15–17 years, a second signature of the interviewer was obtained documenting assent of the participating minor and consent of the parent or guardian. A waiver of documentation of informed consent under 45 CF4 46.117 was approved by the KEMRI and CDC IRBs.

## Results

**Fig 1:**
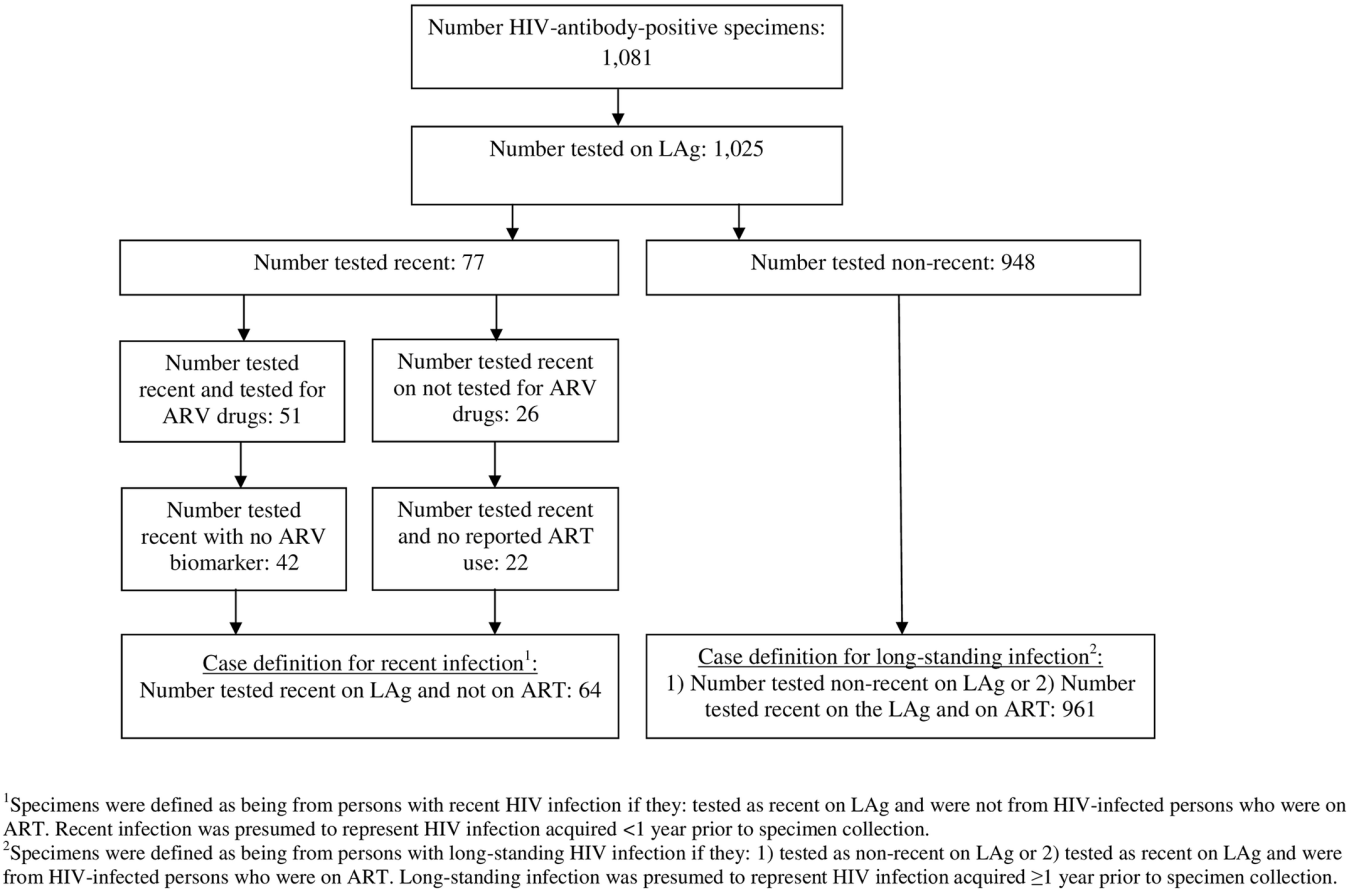
HIV-antibody-positive specimens in the 2007 Kenya AIDS Indicator Survey.

In the 2007 KAIS, 1,081 of 15,583 survey participants with blood specimens for HIV testing were HIV-antibody-positive, and 1,025 (94.8%) of these were available for LAg testing. A total of 77 (7.5%) tested recent and 948 (92.5%) tested non-recent on LAg (Fig 1). Of the 77 specimens that tested recent, 13 were on ART (9 confirmed by ARV drug testing on 51 specimens and 4 confirmed by self-report for 26 specimens not tested for ARV drugs) and were re-defined as long-standing infection in the final analysis. Among HIV-positive specimens tested, the final number of that met the case definition for recent and long-standing infection was 64 (6.2%) and 961 (93.8%), respectively.

### Characterization of recent infection

Table 1 describes the distribution of demographic, behavioral, and biologic variables among individuals with recent and long-standing infection, and Table 2 describes the distribution of these variables among individuals with no HIV infection. The median CD4+ T-cell count was nearly two times as high among recent infections as among long-standing infections [952 cells/mm^3^ (IQR 609–1,243) compared to 483 cells/mm^3^ (IQR 292–721), respectively; p<001]. The majority of recent infections were among women (61.9%), for whom recent infections peaked in the youngest age groups, with 43.0% of recent infection among women aged 15–24 years and 41.6% among those aged 25–34 years. Among men, recent infections were highest in those aged 25–34 years, at 51.0%. A large proportion of recent infections were among participants from rural areas (76.3%) and among persons living in Rift Valley (34.4%) and Nyanza (23.9%) provinces.

**Table 1:** Frequencies and weighted proportions of select demographic, behavioral, and biologic variables among HIV-infected women and men aged 15–64 years by recent and long-standing infection status, 2007 Kenya AIDS Indicator Survey.

	Recent Infection^1^						Long-standing Infection^2^					
	Total (N = 64)		Men (N = 27)		Women (N = 37)		Total (N = 961)		Men (N = 307)		Women (N = 654)	
	N	% (95% CI)	N	% (95% CI)	N	% (95% CI)	N	% (95% CI)	N	% (95% CI)	N	% (95% CI)
**Select variables**												
**Median CD4+ T-cell count (IQR)**^3^		952 (609–1,243)		888 (597–1,133)		977 (670–1,283)		483 (292–721)		435 (264–680)		508 (304–734)
**Sex**												
Men	27	38.1 (22.6–53.7)	-	-	-	-	307	30.6 (27.5–33.8)	-	-	-	-
Women	37	61.9 (46.3–77.4)	-	-	-	-	654	69.4 (66.2–72.5)	-	-	-	-
**Age category (years)**												
15–24	18	29.1 (17.4–40.9)	3	6.6 (0–15.1)	15	43.0 (25.7–60.3)	175	16.3 (13.7–19.0)	26	7.5 (4.3–10.6)	149	20.3 (16.6–24.0)
25–34	26	45.2 (29.8–60.5)	12	51.0 (25.3–76.7)	14	41.6 (23.9–59.2)	344	36.5 (33.1–39.8)	99	34.2 (28.2–40.2)	245	37.5 (33.3–41.7)
35–44	9	12.8 (3.5–22.2)	4	17.9 (0–37.2)	5	9.8 (0.7–18.8)	250	27.7 (24.1–31.3)	103	35.1 (28.7–41.6)	147	24.5 (20.6–28.4)
45–54	7	7.0 (1.5–12.6)	5	11.7 (0–23.5)	2	4.1 (0–9.9)	155	15.6 (13.0–18.1)	62	18.2 (13.4–22.9)	93	14.4 (11.4–17.5)
55–64	4	5.9 (0–12.2)	3	12.8 (0–28.6)	1	1.6 (0–4.6)	37	3.9 (2.5–5.3)	17	5.1 (2.5–7.7)	20	3.4 (1.7–5.0)
**Education**												
No primary	11	16.9 (1.3–32.5)	4	6.4 (0–14.1)	7	23.4 (0.6–46.1)	94	10.9 (8.0–13.8)	12	4.9 (1.5–8.3)	82	13.6 (9.8–17.4)
Incomplete primary	27	45.4 (29.0–61.8)	10	50.3 (24.5–76.1)	17	42.4 (21.9–62.9)	315	32.8 (29.0–36.7)	80	25.7 (19.8–31.6)	235	36.0 (31.2–40.8)
Complete primary	11	15.2 (6.0–24.5)	5	17.2 (0–34.9)	6	14.0 (2.7–25.4)	280	28.7 (25.4–32.0)	91	29.2 (23.0–35.4)	189	28.5 (24.5–32.4)
Secondary+^4^	15	22.4 (9.4–35.5)	8	26.1 (5.3–46.9)	7	20.2 (4.1–36.2)	272	27.5 (23.6–31.4)	124	40.2 (33.6–46.8)	148	22.0 (17.6–26.3)
**Wealth index**^5^												
Lowest	15	24.2 (8.4–40.1)	5	15.7 (0.9–30.5)	10	29.5 (7.4–51.6)	159	16.8 (13.5–20.1)	46	15.3 (10.5–20.2)	113	17.5 (13.8–21.1)
Second	12	17.3 (6.9–27.7)	6	19.1 (0–39.5)	6	16.2 (3.9–28.5)	190	20.1 (16.9–23.4)	57	21.2 (15.9–26.5)	133	19.7 (16.2–23.1)
Middle	10	19.6 (6.1–33.1)	3	15.7 (0–34.6)	7	22.0 (5.0–39.0)	173	17.8 (14.9–20.8)	59	17.9 (12.9–23.0)	114	17.8 (14.5–21.1)
Fourth	13	15.5 (6.2–24.8)	5	14.1 (1.6–26.6)	8	16.4 (4.4–28.4)	189	20.8 (17.1–24.5)	54	17.6 (12.4–22.7)	135	22.2 (17.8–26.6)
Highest	14	23.3 (7.8–38.9)	8	35.5 (6.5–64.5)	6	15.9 (2.0–29.7)	250	24.4 (19.8–29.0)	91	28.0 (21.3–34.7)	159	22.9 (18.0–27.7)
**Residence**												
Urban	15	23.7 (8.1–39.2)	8	33.8 (4.6–63.0)	7	17.4 (3.2–31.6)	299	28.0 (23.5–32.6)	97	25.5 (19.3–31.8)	202	29.2 (23.7–34.6)
Rural	49	76.3 (60.8–91.9)	19	66.2 (37.0–95.4)	30	82.6 (68.4–96.8)	662	72.0 (67.4–76.5)	210	74.5 (68.2–80.7)	452	70.8 (65.4–76.3)
**Province**												
Nairobi	10	13.8 (2.8–24.8)	5	12.8 (0–28.9)	5	14.4 (0.8–28.0)	127	12.5 (8.8–16.1)	42	12.1 (6.7–17.4)	85	12.6 (8.5–16.8)
Central	5	7.8 (0.9–14.7)	0	-	5	12.6 (1.6–23.6)	61	6.8 (3.9–9.6)	22	8.6 (3.4–13.7)	39	5.9 (3.5–8.4)
Coast	9	7.7 (2.1–13.3)	5	11.0 (0.8–21.2)	4	5.7 (0–11.6)	115	9.1 (6.7–11.5)	37	8.3 (5.1–11.5)	78	9.4 (6.4–12.5)
Eastern	4	7.5 (0–15.1)	2	9.8 (0–24.5)	2	6.1 (0–14.5)	95	11.2 (7.6–14.8)	18	6.9 (3.4–10.4)	77	13.1 (8.7–17.6)
North Eastern	1	0.3 (0.2–0.4)	1	0.8 (0–2.4)	0	-	6	0.2 (0–0.5)	3	0.3 (0–0.8)	3	0.2 (0–0.4)
Nyanza	18	23.9 (12.7–35.1)	6	20.6 (4.4–36.8)	12	25.9 (10.7–41.0)	319	32.3 (28.2–36.3)	105	33.8 (27.5–40)	214	31.6 (27.3–35.9)
Rift Valley	13	34.4 (14.0–54.8)	7	42.2 (12.3–72.2)	6	29.6 (5.1–54.1)	135	18.9 (14.8–22.9)	47	19.6 (14.7–24.5)	88	18.5 (13.8–23.3)
Western	4	4.6 (0–9.4)	1	2.7 (0–8.2)	3	5.7 (0–12.6)	103	9.1 (6.7–11.5)	33	10.5 (6.2–14.8)	70	8.5 (6.0–10.9)
**Marital status**												
Never married/cohabiting	7	8.8 (1.9–15.7)	2	3.6 (0–9.4)	5	12.0 (1.0–22.9)	123	12.3 (9.7–14.8)	43	12.1 (8.5–15.8)	80	12.3 (8.9–15.8)
Separated/divorced	3	2.5 (0–5.9)	1	0.4 (0–1.1)	2	3.8 (0–9.2)	107	11.1 (8.5–13.8)	18	5.5 (2.6–8.3)	89	13.6 (10.1–17.1)
Widowed	4	5.0 (0–10.1)	1	2.5 (0–7.5)	3	6.5 (0–14.1)	139	15.5 (12.6–18.4)	15	5.2 (2.7–7.8)	124	20.0 (16.3–23.8)
Currently married/cohabiting	50	83.7 (74.7–92.8)	23	93.5 (85.6–100)	27	77.7 (63.7–91.7)	592	61.1 (57.0–65.2)	231	77.2 (71.9–82.4)	361	54.0 (49.1–59.0)
**Currently pregnant**												
No	33	90.7 (81.7–99.8)	-	-	33	90.7 (81.7–99.8)	611	94.0 (92.2–95.8)	-	-	611	94.0 (92.2–95.8)
Yes	4	9.3 (0.2–18.3)	-	-	4	9.3 (0.2–18.3)	43	6.0 (4.2–7.8)	-	-	43	6.0 (4.2–7.8)
**No. sex partners in past 12 months**												
0–1	54	82.4 (69.0–95.8)	18	58.5 (30.4–86.6)	36	97.1 (91.5–100)	870	91.7 (90.0–93.4)	244	80.2 (75.6–84.8)	626	96.8 (95.4–98.1)
2+	10	17.6 (4.2–31.0)	9	41.5 (13.4–69.6)	1	2.9 (0–8.5)	86	8.3 (6.6–10.0)	63	19.8 (15.2–24.4)	23	3.2 (1.9–4.6)
**Ever used a condom**												
No	45	78.4 (66.4–90.5)	18	72.9 (53.4–92.4)	27	82.0 (66.5–97.5)	553	60.4 (56.6–64.1)	167	57.9 (51.0–64.7)	386	61.5 (57.1–65.8)
Yes	16	21.6 (9.5–33.6)	8	27.1 (7.6–46.6)	8	18.0 (2.5–33.5)	391	39.6 (35.9–43.4)	133	42.1 (35.3–49.0)	258	38.5 (34.2–42.9)
**Condom use at last sex in past 12 months**												
Used a condom	3	7.8 (0–21.3)	2	19.6 (0–50.4)	1	0.3 (0–0.8)	145	15.8 (12.8–18.7)	59	19.8 (15.0–24.5)	86	14.0 (10.6–17.4)
Did not use a condom	58	92.2 (78.7–100)	24	80.4 (49.6–100)	34	99.7 (99.2–100)	799	84.2 (81.3–87.2)	241	80.2 (75.5–85.0)	558	86.0 (82.6–89.4)
**Unprotected sex at last sex with partner of unknown of discordant HIV status in past 12 months**												
No	5	12.0 (0–27.0)	3	22.7 (0–53.2)	2	4.0 (0–11.4)	167	24.0 (20.1–27.9)	68	26.3 (20.7–31.8)	99	22.7 (17.7–27.6)
Yes	50	88.0 (73.0–100)	23	77.3 (46.8–100)	27	96.0 (88.6–100)	576	76.0 (72.1–79.9)	201	73.7 (68.2–79.3)	375	77.3 (72.4–82.3)
**Ever tested for HIV**												
No	40	63.5 (47.6–79.3)	19	68.3 (39.5–97.0)	21	60.3 (42.1–78.5)	511	54.2 (50.2–58.3)	189	64.6 (58.4–70.7)	322	49.7 (44.7–54.6)
Yes	23	36.5 (20.7–52.4)	8	31.7 (3.0–60.5)	15	39.7 (21.5–57.9)	442	45.8 (41.7–49.8)	116	35.4 (29.3–41.6)	326	50.3 (45.4–55.3)
**Self-reported HIV status based on last HIV test result**												
HIV-positive	1	1.2 (0–3.6)	1	3.0 (0–9.0)	0	-	154	17.9 (14.3–21.4)	45	15.4 (10.5–20.4)	109	18.9 (14.8–23.0)
HIV-negative	22	35.3 (19.6–51.1)	7	28.7 (0–57.7)	15	39.7 (21.5–57.9)	272	27.1 (23.8–30.3)	63	18.0 (13.3–22.8)	209	31.0 (27.0–34.9)
Unknown, never tested	40	63.5 (47.6–79.3)	19	68.3 (39.5–97.0)	21	60.3 (42.1–78.5)	511	55.1 (51.0–59.2)	189	66.5 (60.3–72.8)	322	50.1 (45.2–55.1)
**Reported sexually transmitted infection diagnosis or symptoms in past 12 months**												
No	57	85.6 (71.9–99.4)	21	67.3 (38.1–96.5)	36	97.0 (91.0–100)	892	92.8 (90.8–94.8)	271	87.7 (83.6–91.8)	621	95.1 (93.0–97.1)
Yes	7	14.4 (0.6–28.1)	6	32.7 (3.5–61.9)	1	3.0 (0–9.0)	69	7.2 (5.2–9.2)	36	12.3 (8.2–16.4)	33	4.9 (2.9–7.0)
**HSV-2 result**												
Negative	36	59.3 (45.3–73.4)	19	77.0 (59.8–94.2)	17	48.5 (31.8–65.1)	153	15.6 (12.9–18.3)	64	20.5 (15.3–25.6)	89	13.4 (10.3–16.6)
Positive	28	40.7 (26.6–54.7)	8	23.0 (5.8–40.2)	20	51.5 (34.9–68.2)	807	84.4 (81.7–87.1)	243	79.5 (74.4–84.7)	564	86.6 (83.4–89.7)
**Syphilis result**												
Negative	63	97.2 (91.8–100)	26	92.7 (78.8–100)	37	100	921	95.6 (93.9–97.3)	285	93.7 (90.5–96.9)	636	96.4 (94.3–98.5)
Positive	1	2.8 (0–8.2)	1	7.3 (0–21.2)	0	-	37	4.4 (2.7–6.1)	20	6.3 (3.1–9.5)	17	3.6 (1.5–5.7)
**Genital sore or ulcer on or near the penis in past 12 months**												
No	25	78.0 (47.7–100)	25	78.0 (47.9–100)		-	286	93.4 (90.3–96.4)	286	93.4 (90.3–96.4)	-	-
Yes	2	22.0 (0–52.3)	2	22.0 (0–52.1)		-	21	6.6 (3.6–9.7)	21	6.6 (3.6–9.7)	-	-
**Male circumcision status**												
Circumcised	4	10.9 (0–22.8)	4	10.9 (0–22.8)		-	133	42.2 (35.3–49.0)	133	42.2 (35.3–49.0)	-	-
Not circumcised	23	89.1 (77.2–100)	23	89.1 (77.2–100)		-	174	57.8 (51.0–64.7)	174	57.8 (51.0–64.7)	-	-

^1^Specimens were defined as being from persons with recent HIV infection if they tested recent on LAg and were not from HIV-infected persons who were on ART. Recent infection was presumed to represent HIV infection acquired less than one year prior to specimen collection.

^2^Specimens were defined as being from persons with long-standing HIV infection if they: 1) tested non-recent on LAg or 2) tested recent on LAg and were from HIV-infected persons who were on ART. Long-standing infection was presumed to represent HIV infection acquired one year or greater prior to specimen collection.

^3^Based on results from 860 (84%) of 1,025 HIV-positive specimens with CD4+ T-cell count results.

^4^Secondary+ includes any years of secondary schooling whether completed or not.

^5^The wealth index was a composite measure of the living standard of a household, calculated using data on a household’s ownership of selected assets, materials used for houshing construction, water access and sanitation facitlities. The wealth index placed household on a contniuous scale of relative weath using principal components anlysis. Individuals were ranked according to the score of the household in which they resided and the sample was divisded into five groups, each with an equal number of indiviuals (quintiles), ranging from the lowest to highest level of wealth.

**Table 2:** Frequencies and weighted proportions of select demographic, behavioral, and biologic variables among HIV-uninfected women and men aged 15–64 years, 2007 Kenya AIDS Indicator Survey.

	HIV-uninfected					
	Total (N = 14,772)		Men (N = 6,449)		Women (N = 8,323)	
	N	% (95% CI)	N	% (95% CI)	N	% (95% CI)
**Select variables**						
**Sex**						
Men	6449	43.4 (42.3–44.4)	-	-	-	-
Women	8323	56.6 (55.6–57.7)	-	-	-	-
**Age category (years)**						
15–24	4932	34.8 (33.6–35.9)	2176	35.6 (34.0–37.3)	2756	34.1 (32.8–35.4)
25–34	3752	24.5 (23.4–25.6)	1530	22.6 (21.3–24.0)	2222	26.0 (24.6–27.3)
35–44	2670	18.4 (17.6–19.3)	1138	18.0 (16.8–19.3)	1532	18.7 (17.7–19.8)
45–54	2060	13.2 (12.5–14.0)	905	13.0 (12.0–14.0)	1155	13.4 (12.6–14.2)
55–64	1358	9.1 (8.4–9.7)	700	10.7 (9.8–11.6)	658	7.8 (7.1–8.5)
**Education**						
No primary	2106	11.4 (10.3–12.6)	591	6.8 (5.6–8.1)	1515	15.0 (13.6–16.4)
Incomplete primary	4143	29.5 (27.9–31.1)	1795	29.6 (27.5–31.6)	2348	29.4 (27.8–31.0)
Complete primary	3487	24.1 (23.0–25.3)	1526	23.8 (22.3–25.2)	1961	24.4 (23.0–25.8)
Secondary+^1^	5036	35.0 (32.9–37.0)	2537	39.8 (37.5–42.2)	2499	31.2 (29.1–33.3)
**Wealth index**^2^						
Lowest	2571	15.4 (13.5–17.2)	1081	15.4 (13.3–17.5)	1490	15.4 (13.6–17.2)
Second	2702	17.7 (16.0–19.5)	1173	18.0 (16.1–20.0)	1529	17.5 (15.8–19.2)
Middle	2855	19.7 (18.2–21.3)	1241	19.6 (17.9–21.3)	1614	19.9 (18.2–21.5)
Fourth	2880	21.2 (19.4–23.0)	1277	21.4 (19.3–23.5)	1603	21.0 (19.2–22.9)
Highest	3764	26.0 (23.1–28.9)	1677	25.7 (22.8–28.5)	2087	26.2 (23.1–29.3)
**Residence**						
Urban	3599	22.9 (19.8–26.0)	1589	22.3 (19.4–25.3)	2010	23.4 (20.0–26.8)
Rural	11173	77.1 (74.0–80.2)	4860	77.7 (74.7–80.6)	6313	76.6 (73.2–80.0)
**Province**						
Nairobi	1673	9.2 (8.0–10.5)	746	8.8 (7.3–10.3)	927	9.6 (8.2–11.0)
Central	2200	14.7 (13.3–16.0)	961	14.5 (13.0–15.9)	1239	14.8 (13.3–16.3)
Coast	1646	7.7 (6.5–8.9)	704	7.7 (6.4–8.9)	942	7.7 (6.4–9.1)
Eastern	2449	18.1 (16.2–19.9)	1072	18.4 (16.0–20.7)	1377	17.8 (16.1–19.5)
North Eastern	746	2.0 (1.7–2.3)	315	2.0 (1.6–2.4)	431	2.0 (1.7–2.3)
Nyanza	2023	14.1 (12.5–15.6)	875	14.2 (12.3–16.1)	1148	14.0 (12.5–15.5)
Rift Valley	2107	22.3 (19.4–25.1)	944	22.5 (19.9–25.2)	1163	22.0 (18.8–25.3)
Western	1928	12.0 (10.6–13.4)	832	11.9 (10.5–13.4)	1096	12.1 (10.5–13.6)
**Marital status**						
Never married/cohabiting	4386	30.1 (28.8–31.3)	2407	38.4 (36.7–40.1)	1979	23.7 (22.3–25.2)
Separated/divorced	806	5.4 (7.8–12.6)	268	4.2 (3.6–4.8)	538	6.3 (5.6–6.9)
Widowed	623	4.4 (11.9–17.30)	72	1.3 (0.9–1.7)	551	6.8 (6.2–7.5)
Currently married/cohabiting	8957	60.1 (58.8–61.4)	3702	56.1 (54.4–57.9)	5255	63.2 (61.7–64.7)
**Currently pregnant**						
No	7856	94.0 (93.3–94.7)	-	-	7856	94.0 (93.3–94.7)
Yes	467	6.0 (5.3–6.7)	-	-	467	6.0 (5.3–6.7)
**No. sex partners in past 12 months**						
0–1	13825	93.9 (93.4–94.4)	5652	87.9 (86.9–88.9)	8173	98.4 (98.0–98.8)
2+	890	6.1 (5.6–6.6)	766	12.1 (11.1–13.1)	124	1.6 (1.2–2.0)
**Ever used a condom**						
No	8998	69.5 (68.1–70.8)	3566	63.8 (62.0–65.5)	5432	73.6 (71.9–75.4)
Yes	3920	30.5 (29.2–31.9)	1996	36.2 (34.5–38.0)	1924	26.4 (24.6–28.1)
**Condom use at last sex in past 12 months**						
Used a condom	1284	9.9 (9.1–10.6)	810	14.7 (13.5–15.9)	474	6.3 (5.5–7.1)
Did not use a condom	11634	90.1 (89.4–90.9)	4752	85.3 (84.1–86.5)	6882	93.7 (92.9–94.5)
**Unprotected sex at last sex with partner of unknown of discordant HIV status in past 12 months**						
No	1457	13.6 (12.5–14.6)	856	17.7 (16.2–19.2)	601	10.3 (9.1–11.4)
Yes	9277	86.4 (85.4–87.5)	3953	82.3 (80.8–83.8)	5324	89.7 (88.6–90.9)
**Ever tested for HIV**						
No	9652	67.1 (65.7–68.5)	4774	75.8 (74.1–77.5)	4878	60.4 (58.8–62.0)
Yes	4755	32.9 (31.5–34.3)	1560	24.2 (22.5–25.9)	3195	39.6 (38.0–41.2)
**Self-reported HIV status based on last HIV test result**						
HIV-positive	18	0.1 (0.1–0.2)	5	0.1 (0–0.1)	13	0.1 (0.1–0.2)
HIV-negative	4576	32.0 (30.6–33.4)	1482	23.2 (21.4–24.9)	3094	38.7 (37.1–40.4)
Unknown, never tested	9652	67.9 (66.5–69.3)	4774	76.8 (75.0–78.5)	4878	61.1 (59.5–62.7)
**Reported sexually transmitted infection diagnosis or symptoms in past 12 months**						
No	14405	97.4 (97.1–97.7)	6219	96.5 (96.0–97.0)	8186	98.1 (97.6–98.5)
Yes	367	2.6 (2.3–2.9)	230	3.5 (3.0–4.0)	137	1.9 (1.5–2.4)
**HSV-2 result**						
Negative	10151	68.4 (67.3–69.5)	4944	76.4 (75.0–77.8)	5207	62.2 (60.8–63.6)
Positive	4479	31.6 (30.5–32.7)	1455	23.6 (22.2–25.0)	3024	37.8 (36.4–39.2)
**Syphilis result**						
Negative	14391	98.4 (98.1–98.7)	6294	98.4 (98.0–98.8)	8097	98.4 (98.1–98.8)
Positive	222	1.6 (1.3–1.9)	97	1.6 (1.2–2.0)	125	1.6 (1.2–1.9)
**Genital sore or ulcer on or near the penis in past 12 months**						
No	6359	98.6 (98.3–99.0)	6359	98.6 (98.3–99.0)	-	-
Yes	90	1.4 (1.0–1.7)	90	1.4 (1.0–1.7)	-	-
**Male circumcision status**						
Circumcised	854	14.1 (12.3–15.9)	854	14.1 (12.3–15.9)	-	-
Not circumcised	5595	85.9 (84.1–87.7)	5595	85.9 (84.1–87.7)	-	-

^1^Secondary+ includes any years of secondary schooling whether completed or not.

^2^The wealth index was a composite measure of the living standard of a household, calculated using data on a household’s ownership of selected assets, materials used for houshing construction, water access and sanitation facitlities. The wealth index placed household on a contniuous scale of relative weath using principal components anlysis. Individuals were ranked according to the score of the household in which they resided and the sample was divisded into five groups, each with an equal number of indiviuals (quintiles), ranging from the lowest to highest level of wealth.

Most recent infections were among persons in married or cohabiting relationships (83.7%) compared to persons who were never married or cohabiting (8.8%), widowed (5.0%), or separated or divorced (2.5%). Among persons with recent infection who were in a married or cohabiting relationship, 16.7% of men and 4.8% of women reported having sexual partners outside of the relationship in the year preceding the survey. Twenty-nine persons with recent infection had linked HIV test results with a spouse in the household. Among those, 0/29 (0%) had a spouse with recent infection, 9/29 (31.0%) had a spouse with long-standing infection, and 20/29 (69.0%) had a spouse who was HIV uninfected. Among 169 married couples who had sero-discordant HIV results in the survey, 12.9% of HIV-infected men and 13.7% of HIV-infected women had recent HIV infection. Among HIV-positive sero-concordant couples (n = 98), 3.6% of HIV-infected men and 4.3% of HIV-infected women had recent infections.

Most recent infections (63.5%) occurred among persons who had never had an HIV test, followed by persons who believed they were HIV-negative based on their last HIV test result (35.3%) but tested HIV-positive in the survey. Among the latter group, 48.2% reported receiving their HIV-negative test result within the last 12 months. The median CD4+ T-cell count in this group was 1,108 cells/mm^3^; 96.9% were married, and 38.2% were women, of whom 28.1% self-reported being pregnant at the time of the survey, and 41.8% had last tested for HIV infection as part of antenatal care within the year preceding the survey.

### Risk factors for recent and long-standing HIV infection

In bivariate analysis, residence in Nairobi, Nyanza, and Rift Valley provinces; being currently married or widowed; reporting any syndrome or diagnosis of sexually transmitted infections (STI) in the past 12 months; and reporting a genital ulcer (men only) were significantly associated with increased odds of recent infection and considered potential risk factors for recent infection (data not shown). After adjusting for demographics, sexual behavior, and biological indicators, factors independently and significantly associated with higher adjusted odds of recent infection, compared to the HIV-uninfected control population, included living in Nairobi (AOR 11.37; CI 2.64–48.87) and Nyanza (AOR 4.55; CI 1.39–14.89) provinces compared with Western province; being widowed (AOR 8.04; CI 1.42–45.50) or currently married (AOR 6.42; CI 1.55–26.58) compared to being never married; having two or more sexual partners in the past 12 months (AOR 2.86; CI 1.51–5.41); not using a condom at last sex in the past 12 months (AOR 1.61; CI 1.34–1.93); reporting STI diagnosis or symptoms of STI in the past 12 months (AOR 1.97; CI 1.05–8.37); and being younger than aged 30 years with the following characteristics: 1) having laboratory-confirmed HSV-2 infection (AOR 8.84; CI 2.62–29.85), 2) being an uncircumcised male (AOR 17.83; CI 2.19–144.90), or 3) having a male genital ulcer (AOR 8.70; CI 2.36–32.08) (Table 3).

**Table 3:** Factors associated with recent and long-term infection among persons aged 15–64 years in Kenya, 2007 Kenya AIDS Indicator Survey.

	Recent infection compared to HIV uninfected	Long-term infection compared to HIV uninfected
	Adjusted OR^1^^,^^2^	Adjusted OR^2^^,^^3^
	(95% CI)	(95% CI)
**Select variables**		
**Sex**		
Men	1.0	1.0
Women	1.35 (0.46–4.00)	0.99 (0.63–1.54)
**Education**		
No primary	1.0	1.0
Incomplete primary	1.14 (0.42–3.07)	1.42 (1.00–2.02)
Complete primary	0.41 (0.13–1.26)	1.29 (0.88–1.88)
Secondary+^4^	0.41 (0.13–1.30)	1.11 (0.74–1.66)
**Residence**		
Urban	1.0	1.0
Rural	1.54 (0.60–3.92)	1.01 (0.66–1.54)
**Province**		
Nairobi	**11.37 (2.64–48.87)**	1.76 (0.99–3.12)
Central	1.80 (0.43–7.58)	0.79 (0.45–1.37)
Coast	2.31 (0.62–8.66)	**1.55 (1.04–2.32)**
Eastern	0.78 (0.16–3.89)	1.03 (0.67–1.57)
North Eastern	0.31 (0.03–3.16)	0.86 (0.34–2.19)
Nyanza	**4.55 (1.39–14.89)**	**2.33 (1.67–3.25)**
Rift Valley	3.35 (0.93–12.07)	1.31 (0.85–2.04)
Western	1.0	1.0
**Marital Status**		
Never married/cohabiting	1.0	1.0
Separated/divorced	2.03 (0.30–13.59)	**1.87 (1.16–3.01)**
Widowed	**8.04 (1.42–45.50)**	**2.83 (1.78–4.45)**
Currently married/cohabiting	**6.42 (1.55–26.58)**	1.07 (0.73–1.58)
**No. sex partners in past 12 months**		
0–1	1.0	1.0
2+	**2.86 (1.51–5.41)**	0.94 (0.69–1.27)
**Ever used a condom**		
No	1.0	1.0
Yes	0.63 (0.31–1.30)	**1.61 (1.34–1.93)**
**Condom use at last sex with partner in past 12 months**		
Used a condom	1.0	1.0
Did not use a condom	**1.61 (1.34–1.93)**	**0.47 (0.36–0.61)**
**Reported STI diagnosis or symptoms in past 12 months**		
No	1.0	1.0
Yes	**1.97 (1.05–8.37)**	**1.89 (1.20–2.97)**
**Age category (<30 years vs. ≥30 years) and HSV2 infection status (HSV-2 negative vs. HSV-2 positive**)		
Aged <30 years/HSV-2 negative	1.56 (0.39–6.28)	**0.38 (0.20–0.75)**
Aged <30 years/HSV-2 positive	**8.84 (2.62–29.85)**	0.59 (0.33–1.06)
**Age category (<30 years vs. ≥30 years) and circumcision status (circumcised vs. uncircumcised**)		
Aged <30 years/circumcised	0.77 (0.24–2.47)	0.76 (0.37–1.55)
Aged <30 years/uncircumcised	**17.83 (2.19–144.90)**	**0.30 (0.15–0.61)**
**Age category (<30 years vs. ≥30 years) and male genital ulcer status (no male genital ulcer vs. male genital ulcer**)		
Aged <30 years/no male genital ulcer	1.58 (0.10–25.11)	0.24 (0.05–1.05)
Aged <30 years/male genital ulcer	**8.70 (2.36–32.08)**	0.10 (0.55–1.70)

^1^Specimens were defined as being from persons with recent HIV infection if they tested recent on LAg and were not from HIV-infected persons who were on ART. Recent infection was presumed to represent HIV infection acquired less than one year prior to specimen collection.

^2^A multinomial logistic regression model was applied, controlling for all variables listed in the table. Bolded estimates reached a significance level of p<0.05 in multinomial logistic regression anlaysis.

^3^Specimens were defined as being from persons with long-standing HIV infection if they: 1) tested non-recent on LAg or 2) tested recent on LAg and were from HIV-infected persons who were on ART. Long-standing infection was presumed to represent HIV infection acquired one year or greater prior to specimen collection.

^4^Secondary+ includes any years of secondary schooling whether completed or not.

In contrast to risk factors for recent infection, compared to HIV-uninfected persons, factors that were independently and significantly associated with increased adjusted odds of long-standing infection were living in Nyanza (AOR 2.33; CI 1.67–3.25) and Coast (AOR 1.55; CI 1.04–2.32) provinces compared to Western province; being separated/divorced (AOR 1.87; CI 1.16–3.01) or widowed (AOR 2.83; CI 1.78–4.45) compared to being never married; having ever used a condom (AOR 1.61; CI 1.34–1.93); and having had a STI diagnosis or STI syndrome in the past 12 months (AOR 1.89; CI 1.20–2.97). Using a condom at last sex in the past 12 months (AOR 0.47; CI 0.36–0.61); being aged <30 years and HSV-2 negative (AOR 0.38; CI 0.20–0.75);; and being an uncircumcised male aged <30 years (AOR 0.30; CI 0.15–0.61) was associated with lower adjusted odds of long-standing infection compared to their counterparts.

### Direct comparison of recent and long-standing infection

In the model that directly compared recent with long-standing infection, persons with recent infection had significantly higher adjusted odds of living in Nairobi province (AOR 8.46; CI 1.28–55.96) compared to Western province; being currently married (AOR 4.64; CI 1.17–18.37) compared to never being married; not using a condom at last sex in the past 12 months (AOR 5.07; CI 2.06–12.48); having two or more sexual partners in the past 12 months (AOR 3.93; CI 1.49–10.35); and being aged <30 years with 1) laboratory-confirmed HSV-2 infection (AOR15.16; CI 4.50–51.03), 2) male genital ulcer (AOR 7.51; CI 2.25–25.11), or 3) being an uncircumcised male (AOR 90.44; CI 7.64–1070.80) compared to persons aged 30 years and older.

## Discussion

The application of a LAg-based RITA in a nationally representative population-based HIV serologic survey in Kenya provided an opportunity to identify risk factors for recently acquired HIV infection, offering empirical evidence for where HIV prevention strategies should target for greater impact on the HIV epidemic. We found significant demographic, behavioral, and biological differences between recent and long-standing infection, supporting the utility of RITAs to distinguish between factors that are associated with long-standing HIV infection with those that are associated with recent infection. We also observed immunological differences between recent and long-standing infection which further support the robustness of this approach.

Because recent and long-standing infections contribute to the full spectrum of HIV disease, some level of overlap is expected between characteristics associated with recent and long-standing infection. RITAs, however, can help to assess the severity of risk shared by both recent and long-standing infections. For example, we found that being widowed was significantly associated with both recent and long-standing infection. Yet differences were observed in the magnitude of association, with the odds of recent infection over two times that of long-standing infection for widowed persons.

In 2007, the risk of recent infection was highest in the two regions in the country with the largest burden of HIV infection: Nairobi and Nyanza provinces. These provinces accounted for 43% of adults living with HIV in the country, the majority of whom were unaware of their infection [20]. Nearly a decade later, the HIV epidemic has continued to severely afflict these two provinces, which were home to the top five counties with the highest burden of HIV cases in the country in 2015 [28]. Given the high level of unrecognized transmission occurring in these regions, strategies to substantially reduce the infectiousness of individuals is an essential prevention strategy to decrease transmission risks in these settings. By targeting HIV testing in geographic regions and sub-populations that will yield higher numbers of HIV-positive persons, treating these individuals, and ensuring that they adhere to their HIV medications, viral suppression can be achieved. Understanding the unique demographic and epidemiological nuances in each of these regions will be important to determine how these prevention and treatment strategies should be designed and where they should be delivered for greatest impact.

Beyond geographical insights, our results corroborate the importance of behavioral and biological factors that contribute concurrently to transmission of HIV infection. Persons reporting multiple sex partners in the past year were more likely to have recent infection than those who did not. Additionally, though not surprisingly, lack of condom use at last sexual intercourse was a risk factor for recent HIV infection. In combination, these high-risk behaviors also promote transmission of STI which directly increase the infectiousness of HIV and susceptibility for HIV acquisition [29]. When used correctly and consistently with sexual partners, the effectiveness of condoms to prevent sexual transmission of HIV and other STI can be as high as 90–95% [30]. Behavioral interventions that help to modify sexual behavior through education and building skills on how to correctly and consistently use condoms, particularly for those with multiple sexual partners, can be successful but require populations to be motivated to change their behaviors and sustain these changes [31]. Recognizing that condom use in stable relationships is difficult, promotion of condoms should be prioritized for HIV-negative persons who are at greatest risk for new infection, such as those in sexual relationships with partners of HIV-positive or unknown HIV status, and HIV-positive persons. However, given the challenges in relying on behavioral change alone for HIV prevention, behavioral interventions should be delivered in combination with other prevention strategies, including evidenced-based bio-medical, structural, and motivational strategies to strengthen the impact of HIV prevention on a larger population level [31].

Our findings also highlight the influence of sociological aspects on transmission of HIV infection. Widowhood has been previously described as a correlate of HIV infection in Kenya, though likely biased from potential death from AIDS of the deceased partner [32,33]. However this is the first instance where widowhood has been described as being associated with recent infection in Kenya. It is possible that sexual rituals related to widowhood may be facilitating new infections in this population. Widow inheritance or widow cleansing, where a male assumes the social or sexual responsibility of female widow after the death of her husband, is a traditional practice is some regions in Kenya, and the use of condoms in these rituals is discouraged [34,35]. Further exploration around sexual practices and cultural expectations of widows is needed to provide more clarity on factors that may be contributing to the spread of HIV infection among widows to provide culturally-sensitive options for risk reduction in this population.

The vast majority of recent infection was detected among individuals who were married. While external sexual partnerships can play an important role in introducing HIV infection into an existing marital relationship, the majority of new infections are likely to be occurring in committed relationships with a partner who comes into the relationship already HIV infected. Given the important contribution of married and cohabiting couples in Kenya’s epidemic, HIV testing and counseling services provided to couples hold great potential for reducing new HIV infections on a population level. Not only will knowledge of one’s personal and partner’s HIV status help to improve risk reduction strategies within partnerships [36,37], it will facilitate linkages into early HIV care and treatment services and programs for prevention of mother-to-child transmission, resulting in substantial reductions in viral load and HIV transmission risk in the population [3]. As of 2012, only one-third of persons who had ever been tested for HIV had tested together with a sexual partner, highlighting an important gap in reaching those who may be at greatest risk for new infection [38]. Increased efforts are needed to improve awareness of couples testing options and expanding approaches such as family testing in health facilities and home-based HIV testing and counseling in the community.

Younger age, genital ulcerative disease (GUD), and lack of male circumcision have been previously reported as risk factors for incident HIV infection in sub-Saharan Africa [2,4,5,28,39–41]. Independently, these factors were not found to be associated with recent HIV infection in our analysis. However, in combination, persons younger than 30 years of age with laboratory-confirmed HSV-2 infection, self-reported GUD, or lack of male circumcision had higher odds of recent infection compared to their older counterparts. These interactions provide insight on the intersection of biologic factors and acquisition of incident HIV infections among young Kenyans and highlight the opportunity for bio-medical strategies that prevent GUD, including those caused by HSV-2 infection, to facilitate parallel reductions in incident HIV infections in youth. Results from several clinical trials have demonstrated encouraging progress in the expansion of bio-medical options for prevention of HSV-2 infection. Data from two male circumcision trials in Rakai, Uganda and Orange Farm, South Africa found that male circumcision decreased the incidence of HSV-2 infection in men by 28% and 55%, respectively [42,43]. Recent data from the Centre for AIDS Programme of Research in South Africa 004 study found that application of Tenofovir gel before and after sexual intercourse can reduce HSV-2 acquisition by 51% among women [44]. Based on this evidence male circumcision among men and Tenofovir among women, coupled with condoms, may be an important consideration for sexually active youth to reduce the spread of GUD and HSV-2 infection, and by extension, acquisition of HIV infection [45].

Our findings were subject to the following limitations. Though the current standard of analysis for estimating assay-derived HIV incidence includes the use of HIV-1 viral load results in a RITA to reduce the occurrence of false-recent cases, the results of viral load testing of KAIS 2007 samples were suggestive of nucleic acid degradation over time and not included in this analysis [46]. The absence of viral load test results presents an important shortcoming given that low viral loads can be a cause of false-recent misclassification on antibody-based HIV incidence assays, such as LAg. Undetectable levels of viral RNA in HIV-infected populations can occur from either sustained ART or survival of a unique subset of persons, known as elite suppressors, with naturally low viral loads in the absence of ART. Although we were able to remove some false-recent misclassification due to low viral load by taking into account exposure to ART in the RITA applied, the impact was likely to be minimal given that ART coverage in Kenya was low in 2007 [20]. The larger concern was our inability to remove persons with long-standing infection and naturally low viral loads from our final sample of recent infection. Though rare, the level of elite suppression is expected to higher in older epidemics due to survival advantage of these individuals. Based on population-level data in East Africa, these individuals could account for as high as 10% of the untreated HIV-infected population in the region [47]. The frequency of false-recent misclassification for these individuals has been reported to be as high as 13% on LAg based on previous work [25], and extrapolation to our sample suggests that up to 20% of recent infections could potentially be false-recent cases due to low viral load in the absence of treatment. In addition, given the genetic diversity of HIV-1 subtypes in Kenya, some of these misclassified cases may be further explained by differential specificity of LAg by HIV-1 subtype. Dominate HIV-1 strains in Kenya are clade A virus, which comprise over 50% of HIV infection, and clade D virus which covers roughly 12% to 22% of infections [48–53]. Clade D HIV has shown to have high levels of false-recent misclassification on antibody-based HIV incidence assays due to deferred antibody maturation and rapid antibody decline [25,26]. Previous studies have reported LAg-false-recent rates as high 9% to 16% in clade D HIV compared to 1.3% to 2.7% in clade A HIV. Although we observed significantly higher odds of recent infection in the high HIV burden provinces of Nyanza and Nairobi, given the potential variability in the geographic distribution of HIV-1 subtypes in these provinces, these associations should be interpreted cautiously.

Though the potential for false-recent cases in our analysis, the actual impact on population estimates of recent infection does not appear to be substantial. Assuming that LAg’s MDRI for subtype A and D infections lies somewhere between 211 days and 273 days [25], our findings predict that 9% to 11% of adult HIV infections were recently acquired in 2007, equivalent to approximately 130,000 to 150,000 incident infections among 1,400,000 adults living with HIV in 2007 [20]. If we assume, as reflected earlier, that 20% of recent infections in our sample were potential false-recent cases, our adjusted population estimates of incident adult infections would be revised to approximately 100,000 to 120,000 infections. However, in spite of this adjustment, our estimates continue to align well with previously published estimates of adult HIV incidence based multiple estimation approaches, which ranged from 0.6% to 0.7% of the population in 2007 or 120,000 to 140,000 incident infections [10].

Our findings were also vulnerable to other factors associated with false-recent classification which could have led to dilution of observed associations with recent infection. First, the drugs selected for ARV testing were based on the standardized first- and second-line regimens available in public health facilities in Kenya in 2007. If an individual was on an ART regimen that did not include any of these four drugs, the ARV biomarker would not have been detected. Second, if adherence to ART was poor, the ARV biomarker may not have been detected given the variable half-lives of the ARV drugs tested. The number of days post ingestion to the ARV drug assay cut-off was 12–28 days for EFV, 8–9 days for NVP, 1.5 days for 3TC, and 1.5–2.5 days for LPV (personal communication, Karen Cohen, University of Cape Town). Third, we relied on self-reported data to confirm ART status for individuals who did not have specimens available for ARV drug testing (33% of HIV-positive specimens with LAg Avidity ODn <1.5). It is possible that some individuals on ART did not disclose ART use during the interview. Due to the small sample size of recent infection in this analysis, only major risk factors were likely to be significantly associated with recent infection. We were also not able to conduct a comprehensive analysis of linked infections among sexual partners which could have provided important insight on whether recent infections were being acquired from spouses or outside partnerships. Finally, we were not able to monitor changes in risk factors over time using data from the second Kenya AIDS Indicator Survey, conducted in 2012, as less than 25 recent infections were identified in that survey [30].

In conclusion, we identified risk factors for recent infection that were epidemiologically plausible and immediately actionable. An accurate RITA that can be applied in a national population-based serologic survey has wide public health reach as quick as accurate demonstrations of declines in new HIV infection can result in millions of lives saved and reductions in HIV treatment costs. As combination prevention strategies aimed to dramatically reduce HIV transmission are scaled-up, the ability to track the leading edge of the epidemic with reliable information on where and among whom new infections are occurring is more relevant than ever. Given the potential for these data to make a large impact on the HIV epidemic, the application of a RITA in national HIV surveys with the ability to identify false-recent cases through measurement of viral load and ART use, should be routinely considered, where feasible, to identify risk factors for recent infection.

## Supporting Information

S1 File KAIS Analysis and Publication Concept Sheet Template. (DOCX)
