# Supplementary material for: Identifying Risk Factors for Recent HIV Infection in Kenya Using a Recent Infection Testing Algorithm: Results from a Nationally Representative Population-Based Survey
Source: PLoS One. 2016 May 19;11(5):e0155498. doi: 10.1371/journal.pone.0155498 (PMC4873043; doi:10.1371/journal.pone.0155498)
Supplement: S1 File — (DOCX) [file pone.0155498.s001.docx]

**KAIS Analysis and Publication Concept Sheet Template**

*Instructions: The concept sheet should be completed, submitted to the KAIS Analysis and Publication Review Committee and approved by the committee for all analysis, manuscripts, abstracts, and/or publications from the KAIS data. Please submit completed concept sheets to the National AIDS and STI Control Program of the Kenya Ministry of Health at* [*head@nascop.or.ke*](mailto:head@nascop.or.ke)*. Once received, the form will be reviewed by the KAIS Analysis and Publication Review Committee for consideration. A final disposition will be sent within one month of submission.*

| **Date submitted to the KAIS Analysis and Publication Review Committee** |  |
| --- | --- |
| **Proposed Title** |  |
| **First Author/Analyst** *Name, title, address, phone number and e-mail address* |  |
| **Proposed co-Authors (if applicable)** |  |
| **Proposed Senior Author (if applicable)** *Responsible for supervision of first author and publication process* |  |
| **Primary Data Analyst** |  |

| **Type of work**  *Check all that apply (To add a check mark, right click on the box and select properties.)* | |  |
| --- | --- | --- |
| Analysis  Manuscript  Abstract  Presentation | |  |
| **Preliminary plans for publishing or presenting (if applicable)**  *Specify the targeted journal or conference for the publication and/or presentation with the submission deadline.* | |  |
| **Targeted Journal/Conference** | **Submission deadline** | |
| 1. |  | |
| 2. |  | |

| Objectives of the proposed study or analysis |  |
| --- | --- |
| 1. | |
| 2. | |
| 3. | |
| Rationale for the proposed study or analysis *Why is the proposed study or* *analysis needed? Describe the relevant background information to support the proposed study or* *analysis.* |  |
|  | |
| **Key References**  *Please add more rows if necessary.* |  |
| 1. | |
| 2. | |
| 3. | |
| 4. | |

| **Proposed analysis plan**  *How will the research question be answered? Describe the anticipated approach and methods to be used in the analysis.* | | |  |
| --- | --- | --- | --- |
|  | | | |
| **Variables of interest** *Specify the variables of interest for this analysis.* | | |  |
|  |  |  | |
|  |  |  | |
|  |  |  | |
|  |  |  | |

| **Estimated Timeline for Completion**  *Specify the estimated date of completion for the following time points.* | |  |
| --- | --- | --- |
| **Time points** | **Estimated date of completion** | |
| **Draft of table shells** |  | |
| **Tables populated** |  | |
| **Draft submitted to co-authors for review (if applicable)** |  | |
| **Draft submitted to clearance (if applicable)** |  | |

| **KAIS Analysis and Publication Review Committee Decision** |
| --- |
| Approved*  Approved with modifications*  Rejected  Deferred |
| **Justification and comments (if applicable)** |
|  |
| **Date of Notification** |

**For publication submissions only: after a notification of approval is received, the Committee will follow-up with first authors on progress of manuscripts. If approved concepts have not progressed to a complete first draft* ***after three months of the approval notification date****, the concept will be rejected to allow for other potential writers to submit an independent concept sheet on the same topic.*
